# Progressive multifocal leukoencephalopathy, bispecific antibodies, and multiple myeloma: a case report and systematic review of the literature

**DOI:** 10.3389/fonc.2026.1935696

**Published:** 2026-10-28

**Authors:** Marina Tzanni, Panagiotis Polyzoidis, Konstantinos Angelopoulos, Anastasia Skafida, Ioanna Moustaka, Athanasia Sepsa, Pinelopi Korkolopoulou, Georgios Velonakis, Lampis C. Stavrinou, Michail Voulgarelis, Ioanna Stergiou, Despina Fotiou

**Affiliations:** 1 Clinic & Laboratory of Pathophysiology, Department of Medicine, School of Health Sciences, National and Kapodistrian University of Athens, Athens, Greece; 2 Neurology Department, Laikon General Hospital, Athens, Greece; 3 First Department of Pathology, National and Kapodistrian University of Athens, Laikon General Hospital, Athens, Greece; 4 Research Unit of Radiology - 2nd Department of Radiology, National and Kapodistrian University of, Athens, Greece; 5 Second Dept. of Neurosurgery, National and Kapodistrian University of Athens, Athens, Greece

**Keywords:** bispecific antibodies, immune reconstitution inflammatory syndrome, immunosuppression, JC virus, myeloma, opportunistic infections, progressive multifocal leukoencephalopathy

## Abstract

**Background:**

Bispecific antibodies (BsAbs) have transformed outcomes in relapsed/refractory multiple myeloma (RRMM) but carry a substantial, sustained infectious burden, including rare cases of progressive multifocal leukoencephalopathy (PML).

**Methods:**

We present an index case of a patient with RRMM treated with BsAbs diagnosed with PML complicated by immune reconstitution inflammatory syndrome (PML-IRIS) and perform a systematic review of the literature contextualizing this case among all published reports of PML and PML-IRIS following BsAb therapy in multiple myeloma (MM).

**Case presentation:**

We report a 65-year-old woman with triple-class-refractory λ light-chain MM who was sequentially treated with the anti-B-cell membrane antigen (BCMA)×CD3 BsAb elranatamab and, subsequently, the anti-GPRC5D×CD3 BsAb talquetamab. During the second cycle of talquetamab, she developed rapidly progressive confusion, visual disturbance, and generalized tonic–clonic seizures. Cerebrospinal fluid JC virus (JCV) polymerase chain reaction (PCR) was negative on two occasions despite a clinical and radiographic course highly suggestive of PML; brain biopsy confirmed PML-IRIS. Despite talquetamab discontinuation, IVIG, mirtazapine, and compassionate-use nivolumab, the patient achieved only transient neurological stabilization and died 2 months after presentation.

**Results:**

A systematic review of the literature identified 16 additional sources describing 29 patients with PML or PML-IRIS following BsAb therapy for MM, confirmed on full-text review to represent distinct individuals with no duplication between reports. BCMA-directed constructs accounted for most implicated agents, and talquetamab was implicated only in patients with prior or concurrent BCMA-directed exposure. PML-IRIS was documented in two of these patients and could not be excluded in a third. Despite uniformly supportive management, PML-attributable mortality was 89% (25/28) among patients with a definitive reported outcome, including the index case.

**Conclusion:**

PML, and PML-IRIS specifically, is an emerging, likely class-level complication of BsAb therapy in MM. Clinicians should maintain a low threshold for neuroimaging and CSF JCV testing. These findings should be interpreted in light of the substantial clinical benefit that BsAbs provide in this heavily pretreated population.

## 1 Introduction

The treatment paradigm for multiple myeloma (MM) is constantly evolving. Bispecific antibodies (BsAbs) have emerged as promising agents that have revolutionized clinical outcomes, yielding durable remissions in patients with refractory or relapsed disease by engaging T cells to plasma cells through various target antigens like B-cell membrane antigen (BCMA) and G-protein-coupled receptor family C group 5 member D (GPRC5D)—inducing tumor-cell lysis independent of native T-cell receptor specificity (1). Four such agents have received regulatory approval for heavily pretreated RRMM: the BCMA×CD3 constructs teclistamab, elranatamab, and linvoseltamab, and the GPRC5D×CD3 construct talquetamab (2–5).

As a stand-alone monotherapy, these agents have produced response rates of 60%–74% in heavily pretreated relapsed/refractory multiple myeloma (RRMM) (2, 6, 7). Pivotal trials of BsAbs have reported median progression-free survival (PFS) ranging from 11.2 to 17.5 months—11.4 months for teclistamab in MajesTEC-1 and 17.2 months for elranatamab in MagnetisMM-3, with extended follow-up—a meaningful improvement over historical outcomes in comparably refractory populations (2) (7) (8, 9). Despite their efficacy, these therapies induce a complex array of early and delayed complications that significantly impact patient outcomes. Beyond cytokine release syndrome (CRS) and immune effector cell-associated neurotoxicity syndrome (ICANS), BsAbs therapy carries a substantial and sustained burden of infection. Pooled analyses report all-grade infection rates of approximately 56%, with grade ≥3 infections in roughly 24% of patients (10). BCMA-directed agents appear to confer a higher infectious risk than non-BCMA-directed agents, with infection-related mortality exceeding rates historically reported across other MM treatment modalities (11). In one large pooled cohort, hypogammaglobulinemia was documented in over 75% of patients, and infection accounted for approximately one-quarter of all reported deaths (12). Clinical trial and retrospective cohort data show that prophylactic immunoglobulin replacement substantially reduces the risk of infection and its severity in patients receiving BsAbs, particularly anti-BCMA BsAbs (13–15). This heightened susceptibility appears multifactorial, reflecting the interplay of host factors (e.g., age, comorbidities, and baseline immune reserve), disease-related immune dysregulation intrinsic to MM, and treatment-related mechanisms—including hypogammaglobulinemia, neutropenia, and progressive T-cell exhaustion (16, 17).

Rare cases of progressive multifocal leukoencephalopathy (PML) have recently been reported in patients treated with BsAbs, linking the profound immunocompromise induced by these therapies to this life-threatening complication. PML is a debilitating, demyelinating disorder of the central nervous system (CNS) caused by reactivation of latent JC polyomavirus (JCPyV), especially in severely immunosuppressed states and impaired cellular immunity (18). Replication under these conditions may lead to viral genome rearrangements that produce neurotropic variants capable of replicating in glial cells and inducing the lysis of oligodendrocytes—the myelin-producing cells of the CNS (18). PML has historically been rare in MM, typically arising after multiple lines of immunosuppressive therapy. Although reported cases remain few, the high morbidity and mortality of PML, together with the expanding and earlier use of BsAbs in MM, supports heightened vigilance for atypical neurologic presentations in this population. A systematic understanding of their infectious complications—including risk stratification, prophylactic strategies, and early recognition of opportunistic processes such as PML—is needed to preserve the survival benefit these agents provide.

Immune reconstitution inflammatory syndrome (IRIS) refers to a paradoxical clinical and radiological deterioration that occurs when partial restoration of immune competence provokes an inflammatory response against JC virus (JCV)-infected CNS tissue (19, 20). PML-IRIS has been most extensively characterized following antiretroviral therapy (ART) initiation in HIV infection (19), but analogous presentations have also been reported in non-HIV patients after withdrawal of immunosuppressive monoclonal antibody therapy (20). When this response precedes or coincides with recognition of the underlying infection, it may “unmask” previously occult PML—revealing subclinical JCV replication rather than reflecting disease newly acquired at the time of immune recovery. Management is similarly poorly defined: corticosteroids are used empirically to attenuate the inflammatory response in severe cases, but optimal dosing and duration remain undefined, and clinical relapse after steroid discontinuation has been documented (19). Although PML-IRIS has not yet been specifically reported and systematically reviewed in BsAb-treated MM patients, the continuous T-cell activation inherent to this therapeutic class raises the theoretical possibility of an analogous inflammatory response around dosing interruptions, warranting vigilance when neurological deterioration deviates from the expected course of untreated PML.

We report a case of spontaneous PML-IRIS arising during sequential BsAb therapy in a patient with RRMM and a systematic review of the literature to raise awareness of this rare but potentially fatal complication of an increasingly utilized drug class.

## 2 Methods and materials

The new index case of PML-IRIS reported is a woman with RRMM who has been diagnosed and treated in the Department of Pathophysiology, General Hospital of Athens “Laiko” since 2016. The case report is prepared in accordance with CARE (CAse REport) guidelines. Written informed consent for publication of clinical details was obtained from the patient’s next of kin, in accordance with institutional and journal requirements.

We also performed a systematic review to identify case reports, case series, trial safety reports, pharmacovigilance publications, and observational studies describing PML and PML-IRIS following administration of T-cell engaging BsAbs in MM. We searched PubMed/MEDLINE, Embase, Scopus, and Google Scholar from inception to 30 June 2026 (the final search date); the Google Scholar search included the first 200 records sorted by relevance. The main search algorithm was the same for the aforementioned databases and included the following terms: (“bispecific antibody” OR “bispecific antibodies” OR BiTE OR “T-cell engager” OR “T cell engager” OR teclistamab OR elranatamab OR talquetamab OR linvoseltamab OR “BCMA” OR “GPRC5D”) AND (“multiple myeloma” OR “plasma cell myeloma” OR “Kahler disease”) AND (“progressive multifocal leukoencephalopathy” OR “progressive multifocal leucoencephalopathy” OR PML OR “JC virus” OR JCV OR “JCPyV” OR “HPyV2” OR “John Cunningham virus”). Gray literature was handled as follows: the reference lists of all included articles and of relevant narrative reviews were hand-searched; conference abstracts (EHA, ASH, and IMS) were screened via the Google Scholar search and the respective society abstract books; and regulatory safety reports (FDA/EMA product labels and periodic safety update summaries for approved BsAbs) were inspected for PML events. Records identified through these routes were subject to the same eligibility criteria and the same duplicate-screening process as database records. Title and abstract screening and subsequent full-text eligibility assessment were performed independently by two reviewers (M.T. and P.P.); disagreements were resolved by discussion, with adjudication by a third, senior reviewer (D.F.) where consensus could not be reached. A Preferred Reporting Items for Systematic Reviews and Meta-Analyses (PRISMA) flow diagram demonstrates the article selection process (**Figure 1**). The four databases yielded 335 records in total (PubMed/MEDLINE 70, Google Scholar 207, Embase 35, and Scopus 23). After removal of 91 duplicate records, 244 records were screened, of which 174 were excluded at the title and abstract level. Of the 70 reports sought for retrieval, 3 could not be obtained and 67 were assessed in full text; 51 were excluded, leaving 16 sources for inclusion. Inclusion criteria included published and unpublished reports describing PML or PML-IRIS occurring after treatment with BsAbs for RRMM, regardless of prior lines of treatment, that contained sufficient clinical detail on diagnosis, timing, radiologic findings, cerebrospinal fluid testing, management of PML/PML-IRIS, and outcome. We included studies published in English and available in full text or as sufficiently detailed abstracts. We excluded non-bispecific exposures, other hematological malignancies than MM, reports without adequate diagnostic confirmation, and duplicate publications.

**Figure 1:**
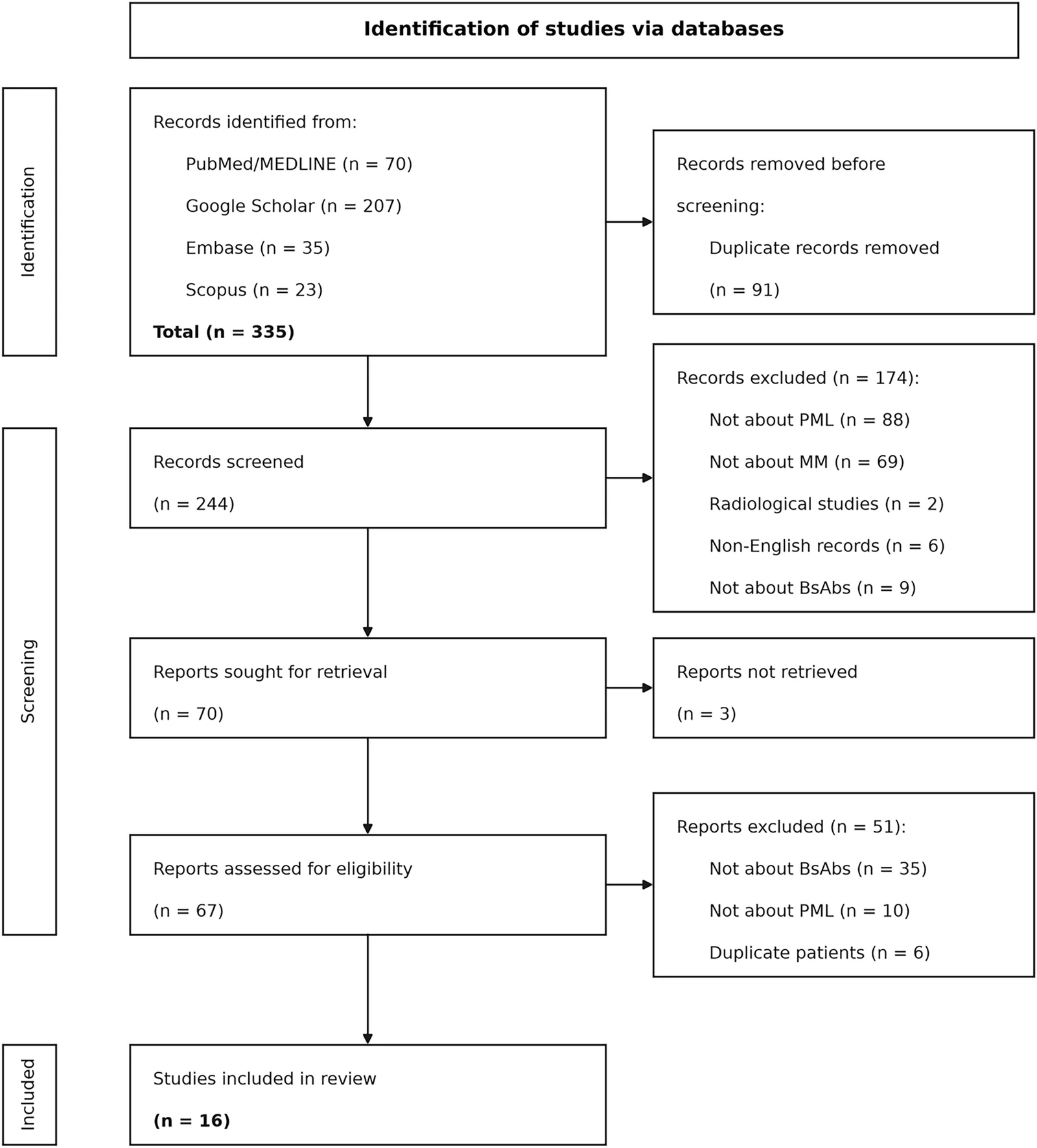
PRISMA flow diagram. BsAbs, bispecific antibodies; MM, multiple myeloma; PML, progressive multifocal leukoencephalopathy.

The methodological quality of included sources was assessed independently by M.T. and P.P. using the tool proposed by Murad et al. for case reports and case series, which evaluates selection, ascertainment, causality, and reporting domains (21). Trial-level safety reports were instead assessed qualitatively for completeness of patient-level detail, as risk-of-bias instruments designed for controlled study designs were not applicable to this single-arm safety reporting format.

## 3 Results

### 3.1 Index case report

#### 3.1.1 Patient information

A 65-year-old woman was diagnosed with λ light-chain MM in 2016, with standard-risk cytogenetics and standard risk by ISS, R-ISS, R2ISS, and IMWG/IMS criteria (22). Past medical history included heterozygous β-thalassemia, hypertension, and psoriasiform dermatitis; family history was unremarkable.

First-line induction with bortezomib, cyclophosphamide, and dexamethasone (VCD) achieved a stringent complete response (sCR), consolidated with autologous stem cell transplantation (ASCT) in 2017 and lenalidomide maintenance. Three years post-ASCT, she developed biochemical relapse and received second-line daratumumab, pomalidomide, and dexamethasone (DPd), achieving a very good partial response (VGPR). One year later, third-line selinexor, carfilzomib, and dexamethasone (SelKd) produced a progressively shortening response, with progressive disease (PD) after approximately 1 year. Her disease became triple-class refractory, with resistance to proteasome inhibitors, immunomodulatory agents, and anti-CD38 monoclonal antibodies.

Fourth-line therapy was the anti-BCMA×CD3 BsAb elranatamab, initiated with step-up dosing in June 2025. Grade 2 CRS was managed successfully with dexamethasone and tocilizumab. A complete response (CR) with negative NGS-based MRD testing was documented after the fourth cycle. Monthly subcutaneous immunoglobulin (scIG) maintained serum IgG, alongside standard antiviral and antifungal prophylaxis.

After biochemical progression on elranatamab (seven cycles), fifth-line therapy with the anti-GPRC5D×CD3 BsAb talquetamab began in February 2026. Grade 1 CRS occurred during step-up dosing; she received two full doses in March 2026.

#### 3.1.2 Clinical findings

While on Cycle 2 (C2) of talquetamab, the patient developed progressive confusion and visual disturbance, followed by a generalized tonic–clonic seizure, requiring hospital admission. On presentation, she was hypertensive and neurological examination revealed disorientation with psychomotor slowing, reduced verbal fluency, occasional paraphasic errors, visual agnosia, and inability to cooperate with formal attention/concentration testing; no focal motor or sensory deficits were identified at this stage. The immune effector cell-associated encephalopathy (ICE) score was 2.

#### 3.1.3 Diagnostic assessment

Initial laboratory evaluation at symptom onset was unremarkable: the complete blood count was within normal limits, without lymphopenia or elevation of acute-phase reactants, and renal and hepatic function were preserved. The CSF leukocyte count was 6 cells/µL (lymphocytic predominance), glucose was within normal limits, and protein was elevated (648 mg/L). CSF polymerase chain reaction (PCR) for varicella-zoster virus, herpes simplex virus 1/2, human herpesvirus 6, and cytomegalovirus were all negative, as were CSF VDRL and serum cryptococcal antigen. Gram stain revealed no organisms or polymorphonuclear leukocytes, and fungal and mycobacterial cultures showed no growth. CSF cytology was negative for malignant cells, and flow cytometry excluded CNS MM.

Non-contrast CT of the brain on hospital day 1 demonstrated a right parieto-occipital hypodense lesion with vasogenic edema, without evidence of acute infarction or haemorrhage, and empiric antiseizure medication was initiated. Subsequent brain magnetic resonance imaging (MRI) revealed multiple areas of T2/fluid-attenuated inversion recovery (FLAIR) hyperintensity with corresponding T1 hypointensity in the subcortical white matter, involving the right frontal and parietal lobes and bilateral occipital regions, with minimal post-contrast enhancement; diffusion-weighted imaging showed increased diffusivity within the lesion core with a rim of reduced diffusion at the interface with deep white matter (**Figure 2**). A routine electroencephalogram (EEG) performed the same day showed diffuse slowing with epileptiform discharges over the right parieto-occipital region, consistent with a nonspecific encephalopathy.

**Figure 2:**
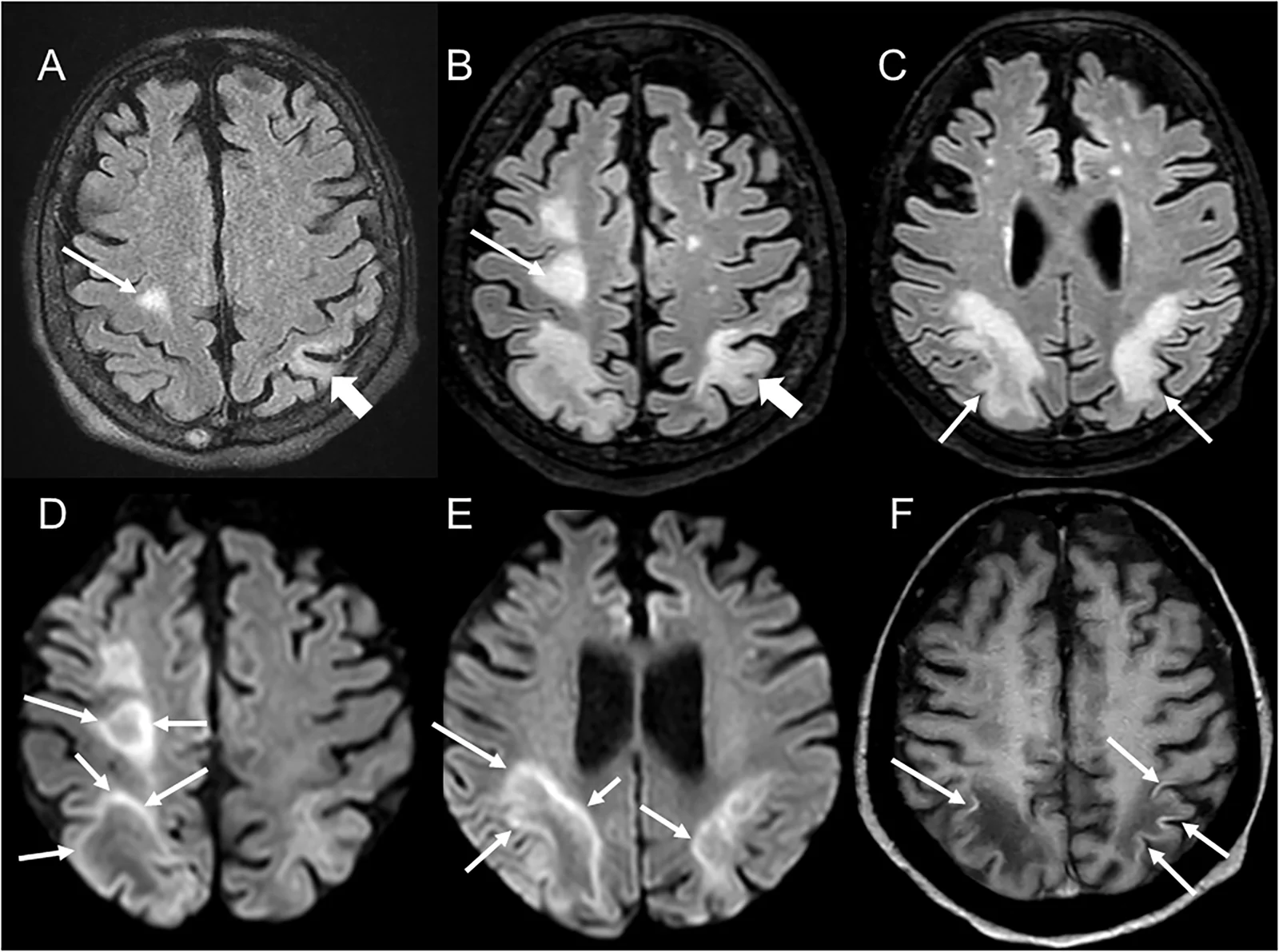
Magnetic resonance imaging. Axial T2–fluid-attenuated inversion recovery (FLAIR) image **(A)** of the initial MRI examination reveals hyperintensities on the right corona radiata (thin arrow A) and on the subcortical white matter of the left postcentral gyrus and inferior parietal lobule (thick arrow A). Axial FLAIR images **(B, C)** from MRI 20 days later show extension of asymmetric bilateral hyperintensities on the corona radiata (thin arrow B), and on bilateral parieto-occipital regions, involving subcortical white matter, including subcortical U-fibers (thick arrow B, thin arrows C). There is no mass effect. Diffusion-weighted imaging (DWI) axial slices **(D, E)** reveal peripheral patchy diffusion restriction, particularly at the leading edge of the lesions (arrows D, E). Findings are highly suggestive of PML. Lesions are hypointense on axial T1-weighted image without contrast administration **(F)**; however, areas of cortical hyperintensity are seen (arrows F), which is a finding associated with seizures complicating PML.

Given this constellation of findings, the differential diagnosis included ICANS, posterior reversible encephalopathy syndrome (PRES), and PML. CSF PCR for JCV was negative. Additionally, a comprehensive autoimmune and paraneoplastic neurological antibody panel was negative. Talquetamab was discontinued, and the patient was empirically treated for presumed grade 3 ICANS with pulse-dose corticosteroids and a 7-day course of anakinra.

Despite these measures, she developed new left hemiparesis, focal pyramidal signs, and ophthalmoplegia by hospital day 15, with persistent epileptiform activity on EEG. Repeat contrast-enhanced black-blood MRI demonstrated interval enlargement of the previously identified white matter lesions and new contrast-enhancing foci in the right frontal lobe (**Figure 2**). Although this clinical and radiological trajectory was highly suggestive of PML, serial CSF JCV PCR remained negative. Given persistently high clinical and radiological suspicion despite negative CSF virology, a stereotactic brain biopsy was performed.

Histopathological examination revealed a demyelinating process with morphological and immunophenotypic features consistent with PML; however, dense perivascular CD8^+^ T-lymphocytic infiltrates raised concern for a concurrent IRIS (PML-IRIS). PCR performed directly on brain tissue confirmed the presence of JCV, establishing the diagnosis. Serum immunoglobulin levels at the time of PML diagnosis were reduced despite scIG administration (IgG 390 mg/dL), and peripheral lymphocyte subset analysis showed a marked reduction in CD3^+^CD4^+^ T lymphocytes, with an inverted CD4^+^/CD8^+^ ratio (0.18). Taken together, three lines of evidence support the classification of this case as PML-IRIS. Clinically, the deterioration was paradoxical in its timing: the new left hemiparesis, focal pyramidal signs, and ophthalmoplegia that emerged by hospital day 15 developed after talquetamab had already been discontinued, that is, at a point when T-cell engagement was being withdrawn rather than sustained. Radiologically, the repeat contrast-enhanced MRI demonstrated not only interval enlargement of the pre-existing white-matter lesions but also new contrast-enhancing foci in the right frontal lobe; enhancement within evolving PML lesions is the accepted imaging correlate of an inflammatory response to JCV-infected tissue (19, 20). Histopathologically, the biopsy demonstrated dense perivascular CD8^+^ T-lymphocytic infiltrates in addition to the demyelinating process and tissue-confirmed JCV, indicating an active cellular immune response within the affected white matter rather than the relative paucity of inflammation that characterizes classical PML.

#### 3.1.4 Therapeutic intervention and outcome

After biopsy confirmation of PML-IRIS, talquetamab—already held since initial presentation—was permanently discontinued. The patient was treated with mirtazapine, selected for its antagonism of the serotonin 5-HT2A receptor implicated in JCV cell entry, and intravenous immunoglobulin (2 g/kg over 3 days). As no clinical improvement was observed and her level of consciousness continued to decline, compassionate, off-label treatment with the anti-PD-1 checkpoint inhibitor nivolumab (3 mg/kg every 2 weeks) was initiated in the absence of established therapeutic options for PML, with the intent of restoring anti-JCV immune surveillance via PD-1 pathway blockade. Two doses of nivolumab were administered during the hospitalization.

Following nivolumab administration, the patient exhibited transient neurological stabilization but did not achieve sustained clinical improvement. Her neurological status subsequently continued to decline, and she died 2 months after her initial hospital admission (**Table 1**).

**Table 1:** Disease timeline.

Time point	Event/Finding
2016	Diagnosis of λ light-chain multiple myeloma
2016	First-line VCD × 6 cycles
2017	Autologous stem cell transplantation; lenalidomide maintenance initiated
2020	Second-line DPd initiated
2021	Third-line SelKd initiated
June 2025	Fourth-line elranatamab initiated
February 2026	Fifth-line talquetamab initiated
March 2026	Two full doses of talquetamab administered
April 2026	Progressive confusion, visual disturbance, generalized tonic–clonic seizure; hospital admission
Hospital day 1	CT brain: right parieto-occipital hypodense lesion with vasogenic edema; ICE score 2; lumbar puncture performed; antiseizure medication started
Hospital day 2	Brain MRI and EEG performed; ICANS/PRES/PML differential considered; CSF JCV PCR negative; autoimmune/paraneoplastic panel negative; talquetamab discontinued; steroid pulses plus 7-day anakinra course started for presumed grade 3 ICANS
Hospital day 15	New left hemiparesis, focal pyramidal signs, ophthalmoplegia, persistent epileptiform EEG; repeat black-blood MRI shows lesion enlargement with new right frontal enhancement
Hospital day 32	Brain biopsy: PML-IRIS; tissue PCR positive for JCV
Post-biopsy	Mirtazapine and IVIG (2 g/kg over 3 days)— no meaningful improvement; progressive decline in consciousness
During hospitalization	Compassionate-use nivolumab (3 mg/kg every 2 weeks) × 2 doses—transient neurological stabilization only
Hospital day 60	Death

CSF, cerebrospinal fluid; CT, computed tomography; DPd, daratumumab, pomalidomide, dexamethasone; EEG, electroencephalogram; ICANS, immune effector cell-associated neurotoxicity syndrome; ICE, immune effector cell-associated encephalopathy (score); IVIG, intravenous immunoglobulin; JCV, JC virus; MRI, magnetic resonance imaging; PCR, polymerase chain reaction; PML, progressive multifocal leukoencephalopathy; PML-IRIS, progressive multifocal leukoencephalopathy–immune reconstitution inflammatory syndrome; PRES, posterior reversible encephalopathy syndrome; SelKd, selinexor, carfilzomib, dexamethasone; VCD, bortezomib, cyclophosphamide, dexamethasone.

### 3.2 Pooled analysis results

Our search identified 16 distinct sources meeting inclusion criteria, describing 29 patients—confirmed on full-text review to represent distinct individuals, with no duplication between reports—with PML occurring in the context of BsAb therapy for MM (**Table 2**): three trial-level safety reports contributing patient-level outcome data without full clinical detail (the linvoseltamab registration trial, *n* = 1; the RedirecTT-1, teclistamab–talquetamab, combination trial, *n* = 1; and the MajesTEC-1 trial, *n* = 1, first reported as a Grade 4/5 adverse event by Moreau et al. and subsequently described in full clinical detail by Hoeynck et al.), 14 patients described across 10 individual case reports or small series, and the 14-patient multimodal registry–pharmacovigilance cohort of Le Hénaff et al. One additional source, a commentary letter by Iltar et al. responding to a review of CAR-T and BsAb toxicities, is included as a bibliographic source discussing the phenomenon; the clinical detail attributed to it in our extraction (a positive CSF JCV PCR) was not accompanied by demographic or treatment data in the same source, and whether it represents an independent patient or a restated example could not be determined from the source. We have retained this patient, as the report describes a distinct clinical event and we found no published case with which it demonstrably overlaps; it is the one patient in this synthesis whose independence rests on the absence of contrary evidence rather than on positive confirmation. We therefore report the pooled outcome estimates with and without this case: excluding it yields 24 deaths among 27 patients with a definitive reported outcome, or 89%, identical to the primary estimate, so that no result or conclusion depends on this judgment. Publications spanned January 2022 to June 2026, with the large majority (12 of 16 sources, 75%) published in 2025 or 2026, consistent with PML’s emergence as a recently recognized complication of this drug class rather than a long-established one.

**Table 2:** Published reports of PML in BsAb-treated patients with MM (case reports, case series, trial safety reports, and registry/pharmacovigilance sources): patient and treatment characteristics.

Case authors, year	Patient number	Age/Sex	MM subtype and stage	LOT	Implicated BsAb (target)	Doses before PML onset	Concurrent infection	Presenting symptoms
Hoeynck et al., 2022 (23); Moreau et al. 2022 (2)	1	50/M	λ LC RRMM	5	Teclistamab (BCMA×CD3)	Multiple (13.6 months on tx before PML)	NR	Gait instability, speech/sensory disturbances
Arvanitis et al. 2024 (24)	1	72/M	κ LC RRMM high-risk cytogenetics (TP53 del)	5	Teclistamab (BCMA×CD3)	1 dose (first step-up: 0.06 mg/kg)	COVID-19	Fever, altered mental status, new right-sided hemiparesis
Chizzoniti et al., 2026 (25)	1	44/F	IgG κ RRMM (biallelic deletion of 1p32)	5	Teclistamab (BCMA×CD3)	C9	NR	Progressive left-sided hemiparesis
Siegel et al., 2026 (31)	1	66/F	IgG κ RRMM	7 (anti-BCMA) 8 (talquetamab)	Anti-BCMA (NR) Talquetamab (GPRC5D×CD3)	C2-talquetamab	COVID-19, CMV, BK virus nephropathy	Progressive decline in mental status, confusion
Cetiner et al., 2026 (28)	1	67/F	κ LC RRMM	6	Elranatamab (BCMA×CD3)	C7	CMV colitis	Dysarthria, psychomotor slowing, gait disturbance, visual impairment, muscle weakness
Tomkins et al. 2026 (29)	1	79/M	κ LC RRMM	5	Elranatamab (BCMA×CD3)	C11	NR	Ataxia, cognitive changes, hemiparesis
Tomkins et al. 2026 (29)	1	70/M	IgG κ RRMM	6	Elranatamab (BCMA×CD3)	C8	CMV encephalitis	Dysphasia, alexia, agraphia
Udovica et al. 2025 (32)	1	69/F	RRMM	8 (teclistamab) 9 (talquetamab)	Teclistamab (BCMA×CD3) Talquetamab (GPRC5D×CD3)	C1-talquetamab	NR	Vertigo, impaired gait, left homonymous hemianopsia
Navarro-Sanchez et al. 2026 (26)	1	51/M	λ LC RRMM (t(11:14))	3	Teclistamab (BCMA×CD3)	C8	NR	Horizontal diplopia, behavioral changes
Haidous et al. 2026 (34)	1	74/M	IgA κ RRMM	5	Cilta-cel following bridging with talquetamab (GPRC5D×CD3)	224 days after infusion of ciltacabtagene autoleucel	NR	Handwriting changes and homonymous right inferior quadrantinopia
Haidous et al. 2026 (34)	1	57/F	κ LC RRMM	6	Anti-BCMA×CD3	C17	NR	Cognitive decline, headaches, and left hemianopia
Iltar et al. 2026 (46)	1	NR	RRMM	NR	Elranatamab (BCMA×CD3)	NR (after multiple cycles)	NR	NR
Bumma et al. 2024 (4)	1	NR	RRMM	≥3	Linvoseltamab (BCMA×CD3)	NR	NR	NR
Cohen et al. 2025 (33)	1	NR	RRMM	≥3	Teclistamab (BCMA×CD3) plus talquetamab (GPRC5D×CD3)	NR	NR	NR
Facchinelli et al., 2026 (30)	1	NR	NR	NR	Elranatamab (BCMA×CD3)	NR	NR	NR
Le Henaff et al. 2026 (27)	14	Median age: 74	RRMM	≥3	BCMA×CD3	Median time: 22 months	NR	Motor/Cognitive impairment, visual symptoms, abnormal gait

ASCT, autologous stem cell transplantation, BsAb, bispecific antibody; BCMA, B-cell maturation antigen; GPRC5D, G-protein-coupled receptor family C group 5 member; IMiD, immunomodulatory drug; JCV, JC virus; LOT, lines of therapy; MM, multiple myeloma; NR, not reported; ORR, overall response rate; PI, proteasome inhibitor; PML, progressive multifocal leukoencephalopathy; RRMM, relapsed/refractory multiple myeloma; *MajesTEC-1 trial PML death: first published Grade 4/5 PML event in a BsAb MM trial (van de Donk et al., *Cancer Cell* 2023).

On methodological quality assessment using the Murad et al. tool (21), the 10 individually described case reports and the Le Hénaff et al. registry–pharmacovigilance cohort satisfied selection and ascertainment criteria, as each explicitly described how patients came to attention and confirmed BsAb exposure through treatment records; reporting was judged adequate in the same sources, with sufficient clinical, radiologic, and, where available, virologic and histologic detail to support independent re-assessment. Causality was more difficult to establish, since alternative explanations for CNS deterioration (e.g., disease progression, CNS infection, and other opportunistic viral processes) were not systematically excluded in the majority of sources, and only three sources reported a biopsy-confirmed diagnosis; this is an inherent limitation of the uncontrolled, retrospective nature of the available evidence rather than a deficiency specific to any individual report. The three trial-level safety reports could not be evaluated with the same instrument, as they lacked the granular clinical detail on which the selection, ascertainment, and causality domains depend, and are instead presented descriptively; this heterogeneity in source quality is itself a limitation of the pooled synthesis and is addressed further below.

BCMA-directed constructs accounted for the substantial majority of implicated exposures. Teclistamab was the most frequently implicated single agent, reported in the Hoeynck et al. (23) case, the Arvanitis and Chizzoniti case reports (24, 25), and the Navarro-Sanchez case report (26), and as the majority exposure (9 of 14 patients, 64%) in the Le Hénaff registry–pharmacovigilance cohort (27).

Elranatamab was implicated in four individually described patients (Cetiner et al.; both patients of Tomkins et al.) (28, 29) and the elranatamab-associated case, without extractable patient-level detail, identified within the broader hematologic–malignancy cohort of Facchinelli et al. (30) and accounted for the remaining 5 of 14 patients (36%) in the Le Hénaff cohort; linvoseltamab was implicated in one patient identified through trial safety reporting (Bumma et al.), the only linvoseltamab-associated case identified in this synthesis (4). Talquetamab (GPRC5D×CD3) was implicated in four patients across the whole synthesis—Siegel et al.’s case (31), Udovica et al.’s case (32), one patient from the teclistamab–talquetamab combination trial (Cohen et al.) (33), and Haidous et al.’s case (34) following bridging therapy before ciltacabtagene autoleucel—but in every instance, only after, or in direct combination with, prior BCMA-directed exposure. No case of talquetamab-associated PML has yet been reported in a BCMA-naive patient.

Presenting features were referable to the affected white-matter territory and included hemiparesis or focal weakness, ataxia and gait disturbance, visual field deficits, dysarthria, dysphasia, alexia or agraphia, and cognitive or behavioral change; no presentation was specific to BsAb exposure as opposed to PML occurring in any other immunosuppressed host. A concurrent viral process—COVID-19, cytomegalovirus (colitis or encephalitis), or BK virus nephropathy—was documented in 4 of 15 sources (Arvanitis: COVID-19; Siegel: COVID-19, CMV, and BK virus nephropathy concurrently; Cetiner: CMV colitis; one of the two Tomkins patients: CMV encephalitis), raising the possibility that intercurrent viral illness lowers the threshold for JCV reactivation in an already immunosuppressed host.

All cases were classified using the 2013 American Academy of Neurology (AAN) consensus criteria for PML (**Table 3**), with the trial-level safety reports (the linvoseltamab trial and the teclistamab–talquetamab trial) classified as “presumed definite” on the basis of a reported Grade 4/5 neurologic adverse event, without the granular diagnostic detail available in the dedicated case reports (35). Where reported, CSF JCV PCR was positive on initial testing in the large majority of individually described cases. Repeatedly negative CSF PCR despite an ultimately confirmed diagnosis was documented in three patients. Both patients described by Tomkins et al. had an initially negative CSF JCV PCR that became positive only on a subsequent lumbar puncture—the second attempt in one patient and the third in the other, by which point the second patient had already developed radiographic and histologic evidence of PML-IRIS. A third patient, who had received a GPRC5D-directed bispecific as bridging therapy before BCMA-directed CAR T-cell therapy, had a CSF JCV PCR that was negative on two occasions and required a stereotactic brain biopsy for a definitive diagnosis (34)—the only biopsy-confirmed case identified in this synthesis. MRI findings were uniformly consistent with classical PML (T2/FLAIR white-matter hyperintensity, typically without mass effect), with a rim of restricted diffusion at the lesion margin noted in a subset. PML-IRIS was explicitly reported in two patients in the reviewed literature (25, 26) and could not be excluded in a third (24).

**Table 3:** Diagnostic and laboratory features of BsAb-Era PML cases in MM.

Case #	JCV CSF PCR (copies/mL)	Brain MRI findings	Brain biopsy	PML diagnosis type (AAN criteria)	PML-IRIS present
Hoeynck et al. 2022 (23)	Positive (NR)	WM lesions T2/FLAIR hyperintense	Not performed	Definite PML (clinical + imaging + CSF JCV+)	NR
Arvanitis et al. 2024 (24)	Positive (quantification NR)	WM lesions T2/FLAIR hyperintense; restricted diffusion	Not performed	Definite PML (clinical + imaging + CSF JCV+)	Cannot exclude
Chizzoniti et al. 2026 (25)	Positive (549)	Subcortical WM lesions T2/FLAIR hyperintense, with corresponding T1 low signal	Not performed	Definite PML (clinical + imaging + CSF JCV+)	Yes
Siegel et al. 2026 (31)	Positive (120,000)	T2 WM hyperintensities lesions T2/FLAIR hyperintense	Not performed	Definite PML (clinical + imaging + CSF JCV+)	NR
Cetiner et al. 2026 (28)	Positive (4.5 × 10^7^)	WM lesions T2/FLAIR hyperintense	Not performed	Definite PML (clinical + imaging + CSF JCV+)	NR
Tomkins et al. 2026 (29)	Positive-second one (NR)	WM lesions T2/FLAIR hyperintense	Not performed	Definite PML (clinical + imaging + CSF JCV+)	NR
Tomkins et al. 2026 (29)	Positive after 2 occasions (NR)	WM lesions T2/FLAIR hyperintense, diffusion-weighted abnormalities	Not performed	Definite PML (clinical + imaging + CSF JCV+)	NR
Udovica et al. 2025 (32)	Positive (NR)	WM lesions T2/FLAIR hyperintense, non-cytotoxic edema	Not performed	Definite PML (clinical + imaging + CSF JCV+)	NR
Navarro-Sanchez et al. 2026 (26)	Positive (NR)	WM lesions T2/FLAIR hyperintense	Not Performed	Definite PML (clinical + imaging + CSF JCV+)	Yes
Haidous et al. 2026 (34)	Negative in 2 occasions	WM lesions T2/FLAIR hyperintense	Performed	Definite PML (clinical + imaging + brain biopsy+)	NR
Haidous et al. 2026 (34)	Positive (637)	WM lesions T2/FLAIR hyperintense	Not performed	Definite PML (clinical + imaging + CSF JCV+)	NR
Iltar et al. 2026 (46)	Positive (NR)	WM lesions T2/FLAIR hyperintense	Not performed	Definite PML (clinical + imaging + CSF JCV+)	NR
Bumma et al. 2024 (4)	NR	NR	NR	Presumed definite (trial-reported Grade 4 AE)	NR
Cohen et al. 2026 (33)	NR	NR	NR	Presumed definite (trial-reported Grade 4 AE)	NR
Facchinelli et al., 2026 (30)	NR	NR	NR	Presumed definite	NR
Le Henaff et al. 2026 (27)	Positive (median load 3, 5 log10)	WM lesions T2/FLAIR hyperintense	Not performed	Definite PML (clinical + imaging + CSF JCV+)	NR

All diagnoses were based on the American Academy of Neurology (AAN) 2013 consensus criteria. AE, adverse event; JCV, JC virus; CSF, cerebrospinal fluid; PCR, polymerase chain reaction; WM, white matter; PML, progressive multifocal leukoencephalopathy; IVIG, intravenous immunoglobulin; CRS, cytokine release syndrome; ICANS, immune effector cell-associated neurotoxicity syndrome; CART, chimeric antigen receptor T-cell therapy; NR, not reported in available publications.

Management was uniformly supportive in intent, reflecting the lack of an approved anti-JCV antiviral (**Table 4**); BsAb therapy was discontinued in essentially every case for which treatment disposition was reported. Beyond discontinuation, adjunctive strategies aimed at restoring anti-JC-virus immune surveillance were common: an immune checkpoint inhibitor was administered to at least 15 of the 29 patients across this synthesis [pembrolizumab in 5 individually described patients and in 8 of the 14 Le Hénaff cohort patients (27); nivolumab in 1 individually described patient (28) and in 1 of the 14 Le Hénaff cohort patients], intravenous immunoglobulin was documented in at least 8 individually described patients, allogeneic JC-virus-specific T-cell transfer was used in 1 patient (32) and 2 of the 14 Le Hénaff cohort patients, and plasmapheresis was used in the 2 patients described by Tomkins et al. (29). Despite these interventions, outcomes were poor: of the patients with a reported definitive outcome, death attributable to PML occurred in the large majority, typically within one to a few months of diagnosis. Only three non-fatal outcomes were identified across the entire synthesis: one patient reported alive and without neurologic progression 32 months after diagnosis (26), and one patient reported improvement and one patient reported stabilization among the 14 patients in the Le Hénaff cohort (27). Outcome data were not reported for two patients: the case of Udovica et al. (32) and the elranatamab-associated patient identified within the broader hematologic–malignancy cohort of Facchinelli et al. (30), for whom no patient-level outcome was extractable. The single linvoseltamab-associated patient reported by Bumma et al. (4) had a fatal outcome (**Table 4**).

**Table 4:** Treatment approaches and clinical outcomes in BsAb-Era PML cases in MM.

Case #	BsAb disposition (at PML diagnosis)	Treatment given for PML	IVIG administered	Corticosteroids used (dose/indication)	Clinical outcome	Time to outcome/follow-up
Hoeynck et al. 2022 (23)	Discontinued	Supportive, pembrolizumab	NR	NR	Fatal	16.1 months from teclistamab initiation; 2.5 months after PML diagnosis
Arvanitis et al., 2024 (24)	Discontinued (after 1 dose)	Supportive	NR	NR	Fatal	NR
Chizzoniti et al., 2026 (25)	Discontinued	Supportive	Yes	NR	Fatal	NR
Siegel et al., 2026 (31)	Discontinued	Supportive	NR	NR	Fatal	2 months from diagnosis of PML
Cetiner et al., 2026 (28)	Discontinued	Supportive, mirtazapine, nivolumab	Yes	NR	Fatal	NR
Tomkins et al., 2026 (29)	Discontinued	Supportive, plasmapheresis, mirtazapine, pembrolizumab	Yes	NR	Fatal	1 month from diagnosis of PML
Tomkins et al., 2026 (29)	Discontinued	Supportive, plasmapheresis, pembrolizumab	Yes	Yes (for CRS/ICANS C1, tocilizumab)	Fatal	2 months after diagnosis of PML
Udovica et al., 2026 (32)	Discontinued	Supportive, pembrolizumab, allogeneic JC virus-specific T-cell therapy	NR	NR	NR	NR
Navarro-Sanchez et al., 2026 (26)	Discontinued	Supportive, pembrolizumab	Yes	NR	Non-fatal	Alive 32 months after PML diagnosis
Haidous et al. 2026 (34)	Discontinued	Supportive, mirtazapine, pembrolizumab	Yes	NR	Fatal	11 months post-CART
Haidous et al. 2026 (34)	Discontinued	Supportive	Yes	NR	Fatal	17 months post- treatment
Iltar et al. 2026 (46)	Discontinued	Supportive	Yes	NR	Fatal	NR
Bumma et al. 2024 (4)	NR	NR	NR	NR	Fatal	NR
Cohen et al. 2026 (33)	NR	NR	NR	NR	Fatal	NR
Facchinelli et al., 2026 (30)	NR	NR	NR	NR	NR	NR
Le Henaff et al. 2026 (27)	Discontinued	Supportive, pembrolizumab, allogeneic JC virus-specific T-cell therapy	NR	NR	12/14 fatal, 1/14 improvement, 1/14 stabilization	1.5 months after PML diagnosis

PML, progressive multifocal leukoencephalopathy; IVIG, intravenous immunoglobulin; CRS, cytokine release syndrome; ICANS, immune effector cell-associated neurotoxicity syndrome; CART, chimeric antigen receptor T-cell therapy; NR, not reported in available publications.

The risk-factor profile was broadly consistent across cases (**Table 5**). Among individually described patients with reported ages, the majority were 65 years or older. Prior exposure to an immunomodulatory agent and an anti-CD38 antibody was reported in essentially all cases with this information available, as was prior ASCT among cases with recorded transplant history, and most patients had received five or more prior lines of therapy. Hypogammaglobulinemia was explicitly documented in roughly half of the sources—very likely an underestimate driven by inconsistent screening rather than a true absence, given how consistently it accompanies BCMA-directed therapy in the broader infection literature.

**Table 5:** Risk factor profile across BsAb-Era PML cases in multiple myeloma.

Case #	Age ≥65	≥5 Prior lines	IMiD exposure	Anti-CD38 exposure	ASCT history	Hypogammaglobulinemia	Concurrent viral infection	BCMA-directed vs. GPRC5D-directed
Hoeynck et al. 2022 (23)	No	Yes	Yes	Yes	NR	Yes	NR	BCMA×CD3 (teclistamab)
Arvanitis et al. 2024 (24)	Yes (72)	Yes	Yes	Yes	Yes	NR	Yes (COVID-19)	BCMA×CD3 (teclistamab)
Chizzoniti et al., 2026 (25)	No	Yes	Yes	Yes	Yes	Yes	NR	BCMA×CD3 (teclistamab)
Siegel et al., 2026 (31)	Yes	Yes	Yes	Yes	Yes	NR	Yes (BKV nephropathy, COVID-19, CMV)	GPRC5D×CD3 (talquetamab) (after prior BCMA BsAb)
Cetiner et al., 2026 (28)	Yes	Yes	Yes	Yes	Yes	Yes	Yes (CMV colitis)	BCMA×CD3 (elranatamab)
Tomkins et al., 2026 (29)	Yes	Yes	Yes	Yes	Yes	Yes	NR	BCMA×CD3 (elranatamab)
Tomkins et al., 2026 (29)	Yes	Yes	Yes	Yes	Yes	Yes	CMV encephalitis	BCMA×CD3 (elranatamab)
Udovica et al., 2025 (32)	Yes	Yes	Yes	Yes	Yes	NR	NR	GPRC5D×CD3 (talquetamab) (after prior teclistamab)
Navarro-Sanchez et al., 2026 (26)	No	No	Yes	Yes	Yes	Yes	NR	BCMA×CD3 (teclistamab)
Haidous et al., 2026 (34)	Yes	Yes	Yes	Yes	Yes	Yes	NR	Cilta-cel following bridging with talquetamab (GPRC5D×CD3)
Haidous et al., 2026 (34)	No	Yes	Yes	Yes	Yes	Yes	NR	BCMA×CD3
Iltar et al. 2026 (46)	NR	NR	NR	NR	NR	Yes	NR	BCMA×CD3 (elranatamab)
Bumma et al., 2024 (4)	NR	NR	Yes	Yes	NR	NR	NR	BCMA×CD3 (linvoseltamab)
Cohen et al., 2026 (33)	NR	NR	Yes	Yes	NR	NR	NR	Teclistamab (BCMA×CD3) plus talquetamab (GPRC5D×CD3)
Facchinelli et al., 2026 (30)	NR	NR	NR	NR	NR	NR	NR	BCMA×CD3 (elranatamab)
Le Henaff et al. 2026 (27)	Yes	Yes	Yes	Yes	Yes	Yes	NR	BCMA×CD3

PML, progressive multifocal leukoencephalopathy; IVIG, intravenous immunoglobulin; CRS, cytokine release syndrome; ICANS, immune effector cell-associated neurotoxicity syndrome; CART, chimeric antigen receptor T-cell therapy; NR, not reported in available publications; BCMA, B-cell maturation antigen; GPRC5D, G-protein-coupled receptor C5D. “Likely” = inferred from disease context or treatment history reported in the case.

## 4 Discussion

BsAbs represent an important therapeutic addition to the MM treatment armamentarium over the last few years, yet their use has introduced a distinct spectrum of infectious complications that remain poorly characterized (1, 10, 36). We undertook a systematic review of the literature, together with the illustrative index case presented above, to characterize PML as an emerging, high-mortality complication of BsAb therapy in MM. Cases have now been reported with every approved BCMA-directed BsAb—teclistamab, elranatamab, and linvoseltamab—and with the GPRC5D-directed agent talquetamab, indicating that this is very likely a class-level phenomenon related to cumulative T-cell engagement and immunosuppression rather than an idiosyncratic effect of a single molecule (37). This interpretation is further corroborated by an independent multimodal study combining a French prospective registry, national pharmacovigilance data, and a global disproportionality analysis in VigiBase^®^, which reported a markedly elevated reporting odds ratio for PML with BsAbs relative to other MM therapies (ROR 17.7, 95% CI 12.1–26.1) and a similarly high case-fatality rate within that cohort alone (86% by 12 months) (27)—closely concordant with the 89% (25/28) PML-attributable mortality observed across the whole of the present synthesis (27). The convergence of case-level and pharmacovigilance-level evidence strengthens confidence that this represents a genuine, if rare, safety signal rather than an artifact of reporting bias alone.

From a mechanistic standpoint, BCMA-directed agents deplete long-lived normal plasma cells, producing deep and often prolonged hypogammaglobulinemia that compounds infection risk (38). GPRC5D-directed agents are hypothesized to have a more favorable infectious profile because GPRC5D is not expressed on B cells or antibody-secreting plasma cells, with emerging data suggesting differential preservation of humoral immunity with anti-GPRC5D compared with anti-BCMA BsAbs (38). The cases identified in this analysis in which talquetamab was implicated all occurred after or alongside BCMA-directed exposure, so that current data cannot yet determine whether GPRC5D-directed therapy carries an intrinsic PML risk independent of prior BCMA exposure.

Several unmet needs emerge from this systematic review and merit particular attention. The first concerns the lack of a validated tool to stratify PML risk after bispecific therapy initiation. Multiple strategies to minimize the infection risk, including antimicrobial prophylaxis, immunoglobulin replacement, vaccination, and reduced dosing frequency have been evaluated and established in standard clinical care (39, 40). No validated tool currently exists to stratify PML risk in patients receiving BCMA- or GPRC5D-directed bispecific therapy for RRMM. This contrasts with natalizumab-associated PML in multiple sclerosis, where anti-JCV antibody serostatus, antibody index, prior immunosuppressant exposure, and treatment duration beyond 24 months have been combined into a validated algorithm that informs monitoring and treatment-continuation decisions (41, 42).

Several features of this population complicate model development. Patients with RRMM are heavily pretreated, having typically received proteasome inhibitors, immunomodulatory agents, anti-CD38 antibodies, and often prior autologous transplantation, each contributing independently to cumulative immunosuppression and confounding attribution of risk to the bispecific agent itself. The T-cell-redirecting mechanism of these agents also produces a distinct immunosuppressive phenotype that may not parallel the mechanisms underlying PML risk models developed in other settings (17). Baseline JCV serostatus is not routinely assessed prior to bispecific initiation in current practice: even recent expert consensus recommendations on infection monitoring during bispecific therapy in myeloma (39, 40, 43) focus largely on hepatitis, HIV, and CMV prevention and treatment and immunoglobulin replacement, without incorporating PML-specific risk assessment.

A closely related unmet need is diagnostic delay and the index of suspicion required to overcome it. The central difficulty is that bispecific therapy produces several syndromes that can mimic or mask PML at presentation. Both CRS and ICANS produce encephalopathy, confusion, and occasionally focal signs in the days after dosing, and clinicians who have been trained to expect these toxicities may anchor on them (44). The European Myeloma Network’s consensus report on adverse-event management is more explicit on one relevant point—that acute, typically self-limited neurotoxicity consistent with ICANS should be distinguished from PML, for which corticosteroids, a mainstay of ICANS management, may be harmful (39, 40, 43). A low threshold for brain MRI and CSF JCV PCR in any BsAb-treated patient with new or progressive neurological symptoms—irrespective of myeloma disease status or timing relative to BsAb initiation—is essential, given that PML occurred in patients in complete response and after more than a year of treatment as well as after a single dose.

A second, arguably more instructive failure mode is false reassurance from a negative initial test. Several published teclistamab-associated cases were confirmed on a single, positive CSF JCV PCR with no repeat lumbar puncture or biopsy required—the first-dose case described above (24) and a later-onset case diagnosed roughly 8 months into therapy despite complete remission (25). The false-negative pattern, however, is also directly documented in myeloma-directed BsAbs. In a UK patient on elranatamab, CSF JCV PCR was negative on two successive lumbar punctures and turned positive only on a third, by which point the patient had already developed PML-IRIS and died within 2 months of diagnosis (29). More strikingly still, in a patient who had received a GPRC5D-directed bispecific together with prior BCMA-directed CAR T-cell therapy, CSF JCV PCR was negative, and the diagnosis was established only by brain biopsy (34). This is further illustrated in our case report.

An equally pressing concern is the uncertain efficacy of immune-reconstitution strategies once PML is diagnosed. Even when the diagnosis is made correctly, there is no reliably effective treatment, and the principal investigational strategy—restoring T-cell function pharmacologically with immune checkpoint inhibitors—has produced inconsistent results that make it difficult to counsel patients or families. This uncertainty is not merely theoretical for bispecific-associated PML: in the largest available cohort, 9 of 14 patients (64%) received a checkpoint inhibitor, yet mortality within that cohort remained 86%, leading its authors to describe the efficacy of immune-reconstitution strategies as “uncertain” despite their “compelling biological rationale and increasing clinical use” (27). This mirrors the broader checkpoint inhibitor literature, in which responses in PML more generally have been inconsistent and appear more closely tied to the underlying cause of immunosuppression than to the checkpoint inhibitor itself (45).

Part of why the field lacks a firm answer to “how much surveillance is enough” is that the underlying driver of infectious risk—T-cell exhaustion—has only recently been framed explicitly as a modifiable target rather than an unavoidable cost of efficacy. Dean’s 2026 review frames continuous BiTE/bispecific exposure as functionally analogous to chronic antigen stimulation: T cells progressively upregulate inhibitory receptors such as PD-1, lose TCF1 expression, and decline in cytokine production, proliferation, and cytotoxicity the longer they are continuously engaged, a pattern reproduced *in vitro* where antigen-specific lysis fell from 88.4% on day 7 to 8.6% by day 28 of continuous BiTE exposure (17). The review’s central proposal is that scheduled treatment-free intervals or fixed-duration dosing can restore T-cell function (*in vitro* lysis recovered to 93.4% with a rest period, versus 34.9% under continuous dosing) without necessarily sacrificing disease control. What is still missing is a prospective, risk-adapted framework for deciding which patients can safely transition to an interval or fixed-duration schedule, and at what point in their disease course (17).

A second, less appreciated unmet need concerns what happens if immune function is subsequently restored—whether through a treatment-free interval, discontinuation of the bispecific after a PML diagnosis, immunoglobulin repletion, or spontaneous T-cell recovery. Sun and Singh’s foundational review of IRIS in non-HIV immunocompromised patients established that IRIS is not unique to antiretroviral-treated HIV: it can occur whenever a sufficiently suppressed immune system recovers function in the presence of a pre-existing, often subclinical, pathogen burden, producing paradoxical clinical deterioration that reflects a restored inflammatory response rather than treatment failure or disease progression (20). The populations they describe as at risk are mechanistically analogous to a bispecific-treated patient whose T-cell engagement is interrupted. For bispecific-associated PML, this creates a second diagnostic trap layered on top of the first one described above: a clinician who successfully raises their index of suspicion, diagnoses PML, and holds the bispecific to allow immune recovery must then also anticipate that the patient may transiently worsen—and needs a way to distinguish PML-IRIS from progressive untreated PML or myeloma relapse, since the management implications (supportive care and, in some non-HIV IRIS contexts, corticosteroids, versus urgent reinstitution of anti-tumor therapy) differ substantially. No bispecific-specific guidance on recognizing or managing this scenario yet exists, which represents a direct extension of the unmet need this discussion opened with (20). .

## 5 Limitations

The systematic review has several important limitations. First, the evidence base consists almost entirely of individual case reports and small case series, which are inherently subject to publication bias favoring more severe or unusual presentations, and precludes reliable estimation of incidence or absolute risk. Second, the possibility of case duplication across independent publications was assessed directly: two reports initially considered potentially overlapping (**Table 2**, cases 1 and 15) were confirmed, on subsequent review of the full texts, to describe two different patients, so that the reported patients represent unique individuals with no duplication between these reports. Residual undetected overlap between the individually reported cases and the multi-patient registry–pharmacovigilance cohort cannot be entirely excluded; similar deduplication challenges have been explicitly addressed in parallel registry-based work (27). Third, one case identified [**Table 2**, case 9; Navarro-Sánchez et al. (26)] is published in a journal not indexed in PubMed or Crossref, and we could not independently corroborate its record against an indexed bibliographic source. We have, however, retained it because it reports sufficient clinical, radiological, and virological detail to meet eligibility criteria; it is the only non-fatal described case and excluding it would raise the pooled PML-attributable mortality from 89% (25/28) to 93% (25/27) rather than lower it. Similarly, the commentary by Iltar et al. (46) is retained, but its independence from previously published cases rests on the absence of contrary evidence rather than on positive confirmation; excluding it leaves the pooled estimate unchanged at 89% (24/27). Fourth, reporting of key variables—including JCV quantification, PML-IRIS status, hypogammaglobulinemia, and detailed treatment sequencing—was frequently incomplete, limiting the granularity of the risk-factor analysis in **Table 5**. Finally, because nearly all reported cases occurred in heavily pretreated, multiply relapsed patients, the findings of this review cannot be directly extrapolated to patients receiving BsAbs earlier in their disease course, in whom immune function may be substantially better preserved.

## 6 Conclusion

Looking ahead, the priority for this field should be risk mitigation rather than treatment avoidance, given the favorable overall benefit–risk balance that BsAbs continue to offer in heavily pretreated MM. Several lines of investigation would meaningfully advance this goal. Prospective, multicenter registries with systematic and mandatory reporting of suspected JCV-related neurological events would address the reporting-bias limitations inherent to the case-level literature synthesized here and permit, for the first time, a defensible estimate of incidence rather than disproportionality alone. JCV serostatus and other candidate biomarkers warrant formal evaluation as pre-treatment risk stratifiers, extending the natalizumab-derived risk-stratification paradigm to a population in which it has not yet been tested. Immune-reconstitution strategies—checkpoint inhibitors and virus-specific T-cell therapy foremost among them—and treatment-free interval or fixed-duration dosing schedules both merit formal, ideally trial-based, assessment rather than continued reliance on uncontrolled case-level experience, given how inconsistent their effect has appeared to date. Finally, clear and widely disseminated clinical pathways to rapidly differentiate PML from ICANS and other bispecific-associated neurotoxicities would directly address the diagnostic delay identified as a central theme of this systematic review.

In summary, PML has emerged as a rare but almost uniformly fatal complication across the class of BCMA- and GPRC5D-directed BsAbs used in MM, arising against a background of profound cumulative disease- and treatment-related immunosuppression. The index case presented here, together with the pooled findings of this literature analysis, underscores the need for heightened clinical vigilance, a low threshold for neuroimaging and CSF JCV testing in any BsAb-treated patient presenting with new neurological symptoms, and coordinated, prospective data collection to better define risk factors and to evaluate emerging immune-reconstitution strategies. These findings should be interpreted within the context of the substantial and well-established clinical benefit that BsAbs continue to provide to patients with heavily pretreated MM.

## Data Availability

The original contributions presented in the study are included in the article/supplementary material. Further inquiries can be directed to the corresponding author.

## Glossary

ASCT: Autologous stem cell transplantation
BCMA: B-cell maturation antigen
BsAbs: Bispecific antibodies
CD: Cluster of differentiation
CMV: Cytomegalovirus
CR: Complete response
CRS: Cytokine release syndrome
CSF: Cerebrospinal fluid
CT: Computed tomography
DPd: Daratumumab, pomalidomide, dexamethasone
EEG: Electroencephalogram
FLAIR: Fluid-attenuated inversion recovery
FLC: Free light chain
GPRC5D: G protein-coupled receptor class C group 5 member D
HHV-6: Human herpesvirus 6
HSV: Herpes simplex virus
ICANS: Immune effector cell-associated neurotoxicity syndrome
ICE: Immune effector cell-associated encephalopathy (score)
IgG: Immunoglobulin G
IRIS: Immune reconstitution inflammatory syndrome
IVIG: Intravenous immunoglobulin
JCV: JC virus
MM: Multiple myeloma
MRD: Measurable residual disease
MRI: Magnetic resonance imaging
NGS: Next-generation sequencing
PCR: Polymerase chain reaction
PD: Progressive disease
PD-1: Programmed cell death protein 1
PML: Progressive multifocal leukoencephalopathy
PRES: Posterior reversible encephalopathy syndrome
RRMM: Relapsed/refractory multiple myeloma
sCR: Stringent complete response
SelKd: Selinexor, carfilzomib, dexamethasone
VCD: Bortezomib, cyclophosphamide, dexamethasone
VDRL: Venereal Disease Research Laboratory (test)
VGPR: Very good partial response

## References

[1] Lee H Neri P Bahlis NJ . Bcma- or gprc5d-targeting bispecific antibodies in multiple myeloma: efficacy, safety, and resistance mechanisms. Blood. (2024) 143:1211–7. doi: 10.1182/blood.2023022499 38194680

[2] Moreau P Garfall AL van de Donk N Nahi H San-Miguel JF Oriol A . Teclistamab in relapsed or refractory multiple myeloma. N Engl J Med. (2022) 387:495–505. doi: 10.1056/nejmoa2203478 35661166 PMC10587778

[3] Bahlis NJ Costello CL Raje NS Levy MY Dholaria B Solh M . Elranatamab in relapsed or refractory multiple myeloma: the MagnetisMM-1 phase 1 trial. Nat Med. (2023) 29:2570–6. doi: 10.1038/s41591-023-02589-w 37783970 PMC10579053

[4] Bumma N Richter J Jagannath S Lee HC Hoffman JE Suvannasankha A . Linvoseltamab for treatment of relapsed/refractory multiple myeloma. J Clin Oncol. (2024) 42:2702–12. doi: 10.1200/jco.24.01008 38879802 PMC11272139

[5] Chari A Minnema MC Berdeja JG Oriol A van de Donk N Rodriguez-Otero P . Talquetamab, a T-cell-redirecting GPRC5D bispecific antibody for multiple myeloma. N Engl J Med. (2022) 387:2232–44. doi: 10.1056/nejmoa2204591 36507686

[6] Chari A Krishnan A Rasche L Ye JC Garfall A Popat R . Clinical management of patients with relapsed/refractory multiple myeloma treated with talquetamab. Clin Lymphoma Myeloma Leuk. (2024) 24:665–93:e14. doi: 10.1016/j.clml.2024.05.003 38871558

[7] Lesokhin AM Tomasson MH Arnulf B Bahlis NJ Miles Prince H Niesvizky R . Elranatamab in relapsed or refractory multiple myeloma: phase 2 MagnetisMM-3 trial results. Nat Med. (2023) 29:2259–67. doi: 10.1038/s41591-023-02528-9 37582952 PMC10504075

[8] van de Donk N Moreau A Garfall A Bhutani D Oriol A Nooka A . Long-term follow-up from MajesTEC-1 of teclistamab, a B-cell maturation antigen (BCMA) x CD3 bispecific antibody, in patients with relapsed/refractory multiple myeloma (RRMM). J Clin Oncol. (2023) 41. doi: 10.1200/jco.2023.41.16_suppl.801142852411

[9] Tomasson MH Iida S Niesvizky R Mohty M Bahlis NJ Martinez-Lopez J . Long-term survival and safety of elranatamab in patients with relapsed or refractory multiple myeloma: update from the MagnetisMM-3 study. Hemasphere. (2024) 8:e136. doi: 10.1002/hem3.136 39055646 PMC11269363

[10] Reynolds G Cliff ERS Mohyuddin GR Popat R Midha S Liet Hing MN . Infections following bispecific antibodies in myeloma: a systematic review and meta-analysis. Blood Adv. (2023) 7:5898–903. doi: 10.1182/bloodadvances.2023010539 37467036 PMC10558589

[11] Nath K Shekarkhand T Nemirovsky D Derkach A Costa BA Nishimura N . Comparison of infectious complications with BCMA-directed therapies in multiple myeloma. Blood Cancer J. (2024) 14:88. doi: 10.1038/s41408-024-01043-5 38821925 PMC11143331

[12] Mazahreh F Mazahreh L Schinke C Thanendrarajan S Zangari M Shaughnessy JD . Risk of infections associated with the use of bispecific antibodies in multiple myeloma: a pooled analysis. Blood Adv. (2023) 7:3069–74. doi: 10.1182/bloodadvances.2022009435 36857755 PMC10331406

[13] Raje N Anderson K Einsele H Efebera Y Gay F Hammond SP . Monitoring, prophylaxis, and treatment of infections in patients with MM receiving bispecific antibody therapy: consensus recommendations from an expert panel. Blood Cancer J. (2023) 13:116. doi: 10.1038/s41408-023-00879-7 37528088 PMC10394080

[14] Cani L Scott SA Roberts D Joseph NS Hofmeister CC Gupta VA . Infection risk in 158 patients with relapsed/refractory multiple myeloma treated with bispecific antibodies: a single-center experience. Haematologica. (2026) 111:656–64. doi: 10.3324/haematol.2025.288187 PMC12862324 40905083

[15] Lancman G Parsa K Kotlarz K Avery L Lurie A Lieberman-Cribbin A . Ivig use associated with ten-fold reduction of serious infections in multiple myeloma patients treated with anti-BCMA bispecific antibodies. Blood Cancer Discov. (2023) 4:440–51. doi: 10.1158/2643-3230.bcd-23-0049 37769148 PMC10618720

[16] Mohan M Chakraborty R Bal S Nellore A Baljevic M D'Souza A . Recommendations on prevention of infections during chimeric antigen receptor T-cell and bispecific antibody therapy in multiple myeloma. Br J Haematol. (2023) 203:736–46. doi: 10.1111/bjh.18909 37287117 PMC10700672

[17] Dean EA . T-cell exhaustion due to BiTE therapy in multiple myeloma: mitigating infectious risks through treatment-free intervals. Front Immunol. (2026) 17:1705345. doi: 10.3389/fimmu.2026.1705345 41646858 PMC12867809

[18] Cortese I Reich DS Nath A . Progressive multifocal leukoencephalopathy and the spectrum of JC virus-related disease. Nat Rev Neurol. (2021) 17:37–51. doi: 10.1038/s41582-020-00427-y PMC7678594 33219338

[19] Tan K Roda R Ostrow L McArthur J Nath A . PML-IRIS in patients with HIV infection: clinical manifestations and treatment with steroids. Neurology. (2009) 72:1458–64. doi: 10.1212/01.wnl.0000343510.08643.74 PMC2677476 19129505

[20] Sun HY Singh N . Immune reconstitution inflammatory syndrome in non-HIV immunocompromised patients. Curr Opin Infect Dis. (2009) 22:394–402. doi: 10.1097/qco.0b013e32832d7aff 19483618

[21] Murad MH Sultan S Haffar S Bazerbachi F . Methodological quality and synthesis of case series and case reports. BMJ Evid Based Med. (2018) 23:60–3. doi: 10.1136/bmjebm-2017-110853 29420178 PMC6234235

[22] Avet-Loiseau H Davies FE Samur MK Corre J D'Agostino M Kaiser MF . Erratum: International Myeloma Society/International Myeloma Working Group consensus recommendations on the definition of high-risk multiple myeloma. J Clin Oncol. (2025) 43:2553. doi: 10.1200/jco-25-01367 40561387 PMC13470823

[23] Hoeynck BW Cohen AD Stadtmauer EA Susanibar-Adaniya SP Vogl DT Waxman AJ . Progressive multifocal leukoencephalopathy in multiple myeloma. Eur J Haematol. (2023) 110:322–9. doi: 10.1111/ejh.13909 36465014

[24] Arvanitis P Farmakiotis D Pelcovits A . Progressive multifocal leukoencephalopathy unmasked by teclistamab in a refractory multiple myeloma patient. Curr Oncol. (2024) 31:2670–8. doi: 10.3390/curroncol31050202 38785483 PMC11119787

[25] Chizzoniti D Ciambelli F Latargia ML Canziani L Godi C Pavesi F . Late-onset progressive multifocal leukoencephalopathy after teclistamab in multiple myeloma: extending the timeline of infectious risk. Haematologica. (2026) 111:2171–4. doi: 10.3324/haematol.2025.300331 41709740 PMC13231087

[26] Navarro-Sánchez M1 Casanova-Espinosa M2 López-Muñoz N1 Alonso-Fernández RA1 Martínez-López J . Anti-PD1 treatment of progressive multifocal leucencephalopathy in a patient with multiple myeloma treated with anti-Bcma bispecific antibodies: a case report. J Cancer Res Rep. (2026) 2(2):1–4.

[27] Le Henaff JR Le Guennec L Schiro P Bihan K Gasnault J Stankoff B . Bispecific antibody therapy in multiple myeloma and risk of progressive multifocal leukoencephalopathy. Blood Adv. (2026) 10(16):5734–9. doi: 10.1182/bloodadvances.2026020556 42284702 PMC13508629

[28] Cetiner T Cagla Bilek M Kirezli SA Ucuncu GY Atas U Iltar U . Progressive multifocal leukoencephalopathy associated with elranatamab therapy in relapsed/refractory multiple myeloma. J Oncol Pharm Pract. (2026) 32(6):1177–81. 10781552261417343. doi: 10.1177/10781552261417343 PMC13490664 41604520

[29] Tomkins O Maamari RG Douthwaite S Roy A Bain H Kohli N . Progressive multifocal leukoencephalopathy (PML) in 2 patients treated with elranatamab. Clin Lymphoma Myeloma Leuk. (2026) 26:e631–5. doi: 10.1016/j.clml.2026.01.017 41760504

[30] Facchinelli D Mazzai L Marchesi F Angotzi F Appio L Ortu La Barbera E . Characteristics and outcomes of progressive multifocal leukoencephalopathy in hematologic malignancies: a SEIFEM retrospective review. Am J Hematol. (2026) 101:1402–6. doi: 10.1002/ajh.70301 41896710

[31] Siegel A Reci S Grossman L Gleason C Crary J Song L . Progressive multifocal leukoencephalopathy and BK virus-nephropathy with bispecific antibody therapy in multiple myeloma. Haematologica. (2026) 111:431–5. doi: 10.3324/haematol.2025.288521 PMC12775763 41063705

[32] Udovica S Kohlmann M Ciovica-Oel I Rommer P Tischer-Zimmermann S Hilbe W . Progressive multifocal leukoencephalopathy in a multiple myeloma patients treated with teclistamab and talquetamab Hemasphere. (2025).

[33] Cohen YC Magen H Gatt M Sebag M Kim K Min CK . Talquetamab plus teclistamab in relapsed or refractory multiple myeloma. N Engl J Med. (2025) 392:138–49. doi: 10.1056/nejmoa2406536 39778168

[34] Haidus M El-Amir Z Frame D Pianko MJ Geer TA . Neurotoxicity mimic: progressive multifocal leukoencephalopathy following BCMA-directed CART and bispecific therapiesa neurotoxicity mimic: progressive multifocal leukoencephalopathy following BCMA-directed CART and bispecific therapies. Transplant Cell Ther. (2026) 2(2):S553–4. doi: 10.1016/j.jtct.2025.12.830

[35] Berger JR Aksamit AJ Clifford DB Davis L Koralnik IJ Sejvar JJ . PML diagnostic criteria: consensus statement from the AAN Neuroinfectious Disease Section. Neurology. (2013) 80:1430–8. doi: 10.1212/WNL.0b013e31828c2fa1 PMC3662270 23568998

[36] Dimopoulos MA Terpos E Boccadoro M Moreau P Mateos MV Zweegman S . EHA-EMN evidence-based guidelines for diagnosis, treatment and follow-up of patients with multiple myeloma. Nat Rev Clin Oncol. (2025) 22:680–700. doi: 10.1038/s41571-025-01041-x 40624367

[37] Jourdes A Cellerin E Touzeau C Harel S Denis B Escure G . Characteristics and incidence of infections in patients with multiple myeloma treated by bispecific antibodies: a national retrospective study. Clin Microbiol Infection Off Publ Eur Soc Clin Microbiol Infect Dis. (2024) 30:764–71. doi: 10.1016/j.cmi.2024.02.023 38432433

[38] Jelinek T Zihala D Zabaleta A Kostopoulos IV Soucek O Venglar O . Selective B-cell subset depletion underlies increased infection risk in patients with MM treated with anti-BCMA vs anti-GPRC5D bsAbs. Blood. (2026) 147:1070–82. doi: 10.1182/blood.2025029572 41405507 PMC13014095

[39] Ludwig H Terpos E van de Donk N Mateos MV Moreau P Dimopoulos MA . Prevention and management of adverse events during treatment with bispecific antibodies and CAR T cells in multiple myeloma: a consensus report of the European Myeloma Network. Lancet Oncol. (2023) 24:e255–69. doi: 10.1016/s1470-2045(23)00159-6 37269857

[40] Rodriguez-Otero P Usmani S Cohen AD van de Donk N Leleu X Gallego Perez-Larraya J . International Myeloma Working Group immunotherapy committee consensus guidelines and recommendations for optimal use of T-cell-engaging bispecific antibodies in multiple myeloma. Lancet Oncol. (2024) 25:e205–16. doi: 10.1016/s1470-2045(24)00043-3 38697166

[41] Bloomgren G Richman S Hotermans C Subramanyam M Goelz S Natarajan A . Risk of natalizumab-associated progressive multifocal leukoencephalopathy. N Engl J Med. (2012) 366:1870–80. doi: 10.1097/01.sa.0000422027.26626.8d 22591293

[42] Plavina T Subramanyam M Bloomgren G Richman S Pace A Lee S . Anti-JC virus antibody levels in serum or plasma further define risk of natalizumab-associated progressive multifocal leukoencephalopathy. Ann Neurol. (2014) 76:802–12. doi: 10.1002/ana.24286 25273271 PMC4282070

[43] Rodriguez-Otero P Moreau P Mateos MV Krauth MT Rasche L Kastritis E . European expert recommendations on teclistamab management for relapsed or refractory multiple myeloma. Clin Lymphoma Myeloma Leuk. (2026) 26:e408–20. doi: 10.1016/j.clml.2025.12.003 41577552

[44] Lee DW Santomasso BD Locke FL Ghobadi A Turtle CJ Brudno JN . ASTCT consensus grading for cytokine release syndrome and neurologic toxicity associated with immune effector cells. Biol Blood Marrow Transplant. (2019) 25:625–38. doi: 10.1016/j.bbmt.2018.12.758 30592986 PMC12180426

[45] Boumaza X Bonneau B Roos-Weil D Pinnetti C Rauer S Nitsch L . Progressive multifocal leukoencephalopathy treated by immune checkpoint inhibitors. Ann Neurol. (2023) 93:257–70. doi: 10.1002/ana.26512 PMC10092874 36151879

[46] Iltar U Atas U Yucel OK Salim O Undar L . JC virus reactivation should be considered in late neurotoxicity associated with bispecific antibody therapy: a comment. Comment on Bangolo et al. Comprehensive review of early and late toxicities in CAR T-cell therapy and bispecific antibody treatments for hematologic malignancies. Cancers (Basel). (2026) 17(2):282. doi: 10.3390/cancers18101506 42192868 PMC13204951

